# Prevalence and factors associated with depression among pregnant mothers in the West Shoa zone, Ethiopia: a community-based cross-sectional study

**DOI:** 10.1186/s12991-020-00275-6

**Published:** 2020-04-06

**Authors:** Takele Tiki, Kefyalew Taye, Bereket Duko

**Affiliations:** 1 Department of Psychiatry Nursing, College of Medicine and Health Science, Ambo University, Ambo, Ethiopia; 2 Department of Public Health, College of Medicine and Health Science, Ambo University, Ambo, Ethiopia; 3 Faculty of Health Sciences, College of Medicine and Health Sciences, Hawassa University, Hawassa, Ethiopia; 4 School of Public Health, Curtin University, Perth, Australia

**Keywords:** Depression, Pregnancy, Community, Ethiopia

## Abstract

**Background:**

Depression during pregnancy, the most prevalent mental health problem, can alter fetal development and has important consequences on the offspring’s physical and mental health. Evidence suggests increasing rates of prevalence of depression in low-income settings such as Ethiopia. However, there are a few studies on the topic with inconsistent results. Therefore, the aim of this study was to investigate the prevalence of antenatal depression and its correlates among pregnant women in Ethiopia.

**Methods:**

A community-based cross-sectional study was conducted in the West Shoa zone, Oromia regional state, Ethiopia, from February 20, 2018, to March 20, 2018. Pregnant women were recruited by using cluster sampling techniques. Data on socio-demographic, obstetric, and psychosocial characteristics were collected by interviewer-administered questionnaire. Patient Health Questionnaire (PHQ-9) was used to assess depression during pregnancy. Bivariable and multivariable logistic regression analyses were fitted to identify correlates of depression. The level of statistical significance was declared at *p* value < 0.05.

**Results:**

The mean age (± SD) of the pregnant women was 28.41 ± 5.9 years. The prevalence of depression during pregnancy was 32.3%. When we adjusted for possible confounding variables in the final model; those pregnant mothers with an average monthly income of less than 500 (18 USD) Ethiopian birr [AOR = 3.19, 95% CI (1.47, 6.96)], unplanned pregnancy [AOR = 1.52, 95% CI (1.04, 2.21)] and having history of abortion [AOR = 5.13, 95% CI (2.42, 10.85)] have higher odds of depression when compared to their counterparts.

**Conclusion:**

The prevalence of depression during pregnancy was high. Strengthening the counseling service as well as increasing access and availability of modern contraceptive methods may reduce the rates of unplanned pregnancy in Ethiopia and this, in turn, plays a significant role in alleviating a resultant depression. Further, the integration of mental health services with existing maternal health care as well as strengthening the referral system among public health centers was warranted to minimize antenatal depression in the West Shoa zone health facilities.

## Background

Depression, also known as major depressive disorder, is a mental health problem characterized by at least 2 weeks of depressed mood that is present across most situations and accompanied by low self-esteem, loss of interest in daily activities, low energy, guilty feeling and death wish [1]. According to the World Mental Health Survey, on average 1 in 20 individuals has at least one episode of depression in their lifetime [2]. The World Health Organization (WHO) predicted depression to become the second leading causes of global disability burden by the year 2020 [2]. Further, depression led to a worldwide total of over 50 million Years Lived with Disability (YLD) in 2015 and over 80% of this disease burden occurred in low- and middle-income countries [3, 4].

In developed countries, the prevalence of prenatal depression is estimated to be 720% [5]; whereas, in developing countries 25% [6]. Depression commonly appears during pregnancy in resource-limited settings [6] and approximately one in three women has a significant mental health problem [5]. However, it is left undiagnosed in low- and middle-income countries, making a substantial contribution to maternal and infant morbidity [7, 8]. The type of tools used to screen depressive symptoms, the study design and setting, the trimester of pregnancy, psychosocial, socio-economic and cultural disparities are the commonly reported reasons for the variations of antenatal depression in developed and developing countries [6–10].

Antenatal depression may be related to numerous risk factors and its occurrence suggestively increases during the mid- and late-trimesters [11]. Findings from different studies showed that the associations between antenatal depression and previous history of psychiatric illness, pregnancy-related complications like stillbirth and history of abortion [12]. Further, it is also associated with perinatal outcomes, growth and development of the previous child [13, 14], past history of depression, unplanned pregnancy [15], single marital status, having thoughts of perceived stress [16], lower educational level, suffering and having history of abuse [17].

Evidence from different studies suggests the intergenerational transmission and progression of depression to adulthood [18–20]. A study from the UK assessed the intergenerational transmission of depression from mothers to offspring [20]. In this study, exposure to prenatal depression by age of 12 years predicated the progression of depression in adolescence. Adjusting such potential mediators in the statistical models may increase the robustness of the estimates. Antenatal depression was also associated with the socio-economic status of women. For example, a population-based cohort study conducted in the Netherlands that included a total of 5398 pregnant mothers reported a statistically significant association between antenatal depression and income [21].

In Ethiopia, there were a few studies with inconsistent results. For example, cross-sectional studies conducted to assess the prevalence of antenatal depression in the northern and central Ethiopia reported 11.8% [22] and 31.1% [23], respectively. Besides, most of the studies were conducted in the clinical setting which plays a significant role in the variation of the prevalence of depression in the antenatal period. Therefore, this study aimed to assess the prevalence of depression and identify associated factors among pregnant mothers in the community setting of Ethiopia.

## Methods

### Study design and setting

A community-based cross-sectional study was employed. The study was conducted in the West Shoa zone, Oromia regional state, Ethiopia. The west Shoa zone has a total of 23 woredas (districts) including Ambo town. The projected population of the West Shoa zone is about 2.6 million (1.3 million men and 1.3 women) population. During the study period, there were 26, 7399 pregnant women with 17, 9160 of which are in the 2nd and 3rd trimester of pregnancy. The assessment was conducted from February 20, 2018, to March 20, 2018.

### Sample size estimation and recruitment of participants

Single population proportion formula was used to calculate the sample size using the magnitude of depression in pregnant mothers in Ethiopia, 31.1% [23], with a 95% confidence interval, 5% of margin error and with the calculated design effect of 2.5. A cluster sampling technique was employed to select three woredas, such as Jeldu, Ambo, and Bako out of 23 woredas. Health extension workers of each woreda listed pregnant mothers using the non-identifying registration code. We have used this registration as a sampling frame to recruit the study participants. A total of 874 pregnant mothers who were in the second or third trimester of pregnancy, lived and currently living in the study area for at least the preceding 1 year were recruited for the study. Those with hearing or cognitive impairment to the extent of impairing capacity to communicate adequately and unable to give informed consent to take part in the study were excluded from the study.

### Data collection instrument and data collectors

Data were collected by trained data collectors. The presence of antenatal depression was assessed by the Patient Health Questionnaire item nine (PHQ-9). This scale has been validated to use among pregnant women in the community setting with the minimum cut-off point of 8 or more suggest depression [24]. This is a highly reliable scale with a sensitivity of 80.8% and a specificity of 79.5% to assess depression. Its reliability coefficient, Cronbach’s alpha, and 1-week test–retest were 0.84 and 0.98, respectively. Perceived stress scale was also used in the study. It is a 10-item Likert scale; each item has 5 possible responses measuring the frequency of perceived stress over the last month [25, 26]. These 10 items are to assess stress due to events, feeling out of control, and feeling rushed or short on time. It has been used in different studies conducted in Ethiopia. It was highly reliable in our study with Cronbach’s alpha of 0.92. We have used the Ethiopian Demographic and Health Survey (EDHS) of 2016 formats to assess other pregnancy-related information (like previous stillbirth, spontaneous abortion, neonatal and infant mortality, and comorbid medical conditions, actual antenatal visits and birth preparedness) [27].

### Operational definitions

#### Antenatal depression

A score of 8 or more in the Patient Health Questionnaire item nine (PHQ-9) was considered depression in this study [24].

#### Perceived stress

The scores above mean in perceived stress scale were considered perceived stress in this study [25, 26].

#### Abortion

It is the ending of a pregnancy by removal or expulsion of an embryo or fetus before it can survive outside the uterus. In this study, it includes spontaneous abortion and induced abortion.

### Data analysis

The Statistical Package for Social Science (SPSS) version 21.0 was used for data analysis. Pregnant mothers’ socio-demographic, economic and obstetric characteristics were described using the statistics of frequency and percentage distributions. Further, bivariate logistic regression analysis was conducted to identify correlates of antenatal depression. Variables with a p-value < 0.25 during bivariate analysis were entered into a multivariate logistic regression analysis to identify potential confounders. Then, adjusted OR was calculated using multivariate logistic regression analysis and the level of significance of association was determined. Significance level was declared at  < 0.05.

## Results

### Socio-demographic characteristics of respondents

A total of 862 pregnant mothers were included in the study yielding a response rate of 98.6%. The mean age (± SD) of the pregnant mothers was 28.41 ± 5.9 years. Out of 862 pregnant mothers, 748 (86.8%) were from the Oromo ethnic group, 342 (29.7%) had no formal education, 746 (86.5%) were housewives and 668 (77.5%) were from rural residence area (Table 1).

**Table 1:** Socio-demographic characteristics of the included women (*n* = 862) in West Shoa zone, Ethiopia, 2018

Variables	Frequency	Percentage
Age of respondents		
15–24	215	24.9
25-34	450	52.2
≥35	197	22.9
Ethnicity of respondents		
Oromo	748	86.8
Amhara	62	7.2
Others	52	6.0
Religion of respondents		
Orthodox	458	53.1
Protestant	365	42.3
Others	39	4.5
Educational status		
No formal education	342	39.7
Primary education	371	43.0
Secondary education	103	11.9
Diploma and above	46	5.3
Occupational status		
House wives	99	11.5
Merchant	697	80.9
Others	66	7.7
Monthly family income		
<500 ETB	340	39.4
500–1500 ETB	381	44.2
≥ 1500 ETB	141	16.4
Residence		
Urban	194	22.5
Rural	668	77.5

### Obstetric characteristics

Regarding the obstetric characteristics, 612 (71%) of the pregnant mothers had planned their current pregnancy, 258 (29.9%) of the pregnant mothers had four or more gravidity and 108 (12.5%) had a history of chronic illness. Forty (4.6%) of the pregnant mothers had a history of depression. Further, 50 (5.8%) of the pregnant mothers experienced spontaneous abortion in their previous pregnancy (Table 2).

**Table 2:** Pregnancy-related factors of women (*n* = 862) in West Shoa zone, Ethiopia, 2018

Variables	Frequency	Percentage
Number of gravidity		
< 2	318	36.9
23	286	33.2
> 4	258	29.9
Parity		
Multipara	559	64.8
Primipara	303	35.2
Pregnancy intention		
Intended	612	71.0
Unintended	250	29.0
Place of birth preceding most recent		
Home	526	61.0
Health institution	336	39.0
Mode of delivery		
Spontaneous vaginal delivery	725	84.1
Instrumental delivery	91	10.6
Cesarean section	46	5.3
The most recent birth attended by		
Relative	376	43.6
TBA	150	17.4
Health workers	336	39.0
History of complication during delivery		
Yes	222	25.8
No	640	74.2
History of abortion		
Yes	50	5.8
No	812	94.2
History of still birth		
Yes	99	11.5
No	763	88.5
ANC visit during previously pregnancy		
Yes	448	52.0
No	414	48.0
ANC visit during last pregnancy		
Yes	615	71.3
No	247	28.7
History of chronic illness		
Yes	108	12.5
No	754	87.5
Symptoms of pregnancy complication		
Yes	202	23.4
No	660	76.6
History of depression		
Yes	40	4.6%

### Prevalence and factors associated with antenatal depression

The prevalence of antenatal depression was 32.3%. The following variables were not significantly associated with antenatal depression in the bivariate analysis: ethnic group, religion, marital status, educational status, occupational status, number of gravidity, number of parity, previous place of delivery, mode of delivery, previous complication during birth, history of stillbirth in lifetime, history of chronic illness and age of respondents. However, an average monthly income of the family, planned pregnancy, perceived stress and previous history of abortion were statistically associated with depression in the bivariate analysis. In the multivariable analysis, the prevalence of antenatal depression was significantly higher among women who did not have plan for the current pregnancy. Women who did not have a plan for their current pregnancy were 1.52 times more likely to have antenatal depression than those who planned for their current pregnancy [AOR = 1.52; 95% CI 1.042.21]. The odds of having antenatal depression increases by fivefold in pregnant women who had a previous history of abortion in their lifetime when compared to pregnant women who had no history of abortion in their lifetime [AOR = 5.13; 95% CI 2.4210.85]. Those pregnant women with a low level of income (monthly income of less than 500 ETB) were 3.19 times more likely to develop antenatal depression when compared to those who earned a monthly income of more than 1500 ETB [AOR = 3.19; 95% CI 1.476.96] Table 3.

**Table 3:** Factors associated with depression among pregnant mothers in West Shoa, Ethiopia, 2018 (n = 862)

Variables	Depression		COR (95% CI)	AOR (95% CI)
	Yes	No		
Age of respondents				
15–24 years	62	153	1.00	1.00
25–34 years	136	314	1.07 (0.75, 1.53)	0.63 (0.41, 1.97)
≥ 35 years	80	117	1.69 (1.12, 2.54)	0.88 (0.54, 1.43)
Monthly family income				
< 500	151	189	4.09 (2.49, 6.73)	3.19 (1.47, 6.96)*
500–1500	104	277	1.93 (1.17, 3.18)	1.80 (0.83,3.92)
≥ 1500	23	118	1.00	1.00
Residence				
Urban	40	154	1.00	1.00
Rural	238	430	2.13 (1.45, 3.12)	1.56 (0.95, 2.49)
Pregnancy plan				
Planned	164	436	1.00	1.00
Unplanned	114	148	2.05 (1.51, 2.77)	1.52 (1.04, 2.21)*
Symptoms of pregnancy complication				
Yes	84	113	1.81 (1.30, 2.51)	1.33 (0.89,1.99)
No	194	471	1.00	1.00
History of abortion in life time				
Yes	33	17	4.49 (2.46, 8.22)	5.13 (2.42, 10.85)*
No	245	567	1.00	1.00
Perceived stress				
Yes	86	132	1.53 (1.11, 2.11)	1.29 (0.87,1.88)
No	192	452	1.00	1.00
History of depression				
Yes	22	18	2.70 (0.98, 4.33)	1.33 (0.75, 2.75)
No	256	566	1.00	1.00

Variables that were used for the adjustment: history of abortion, perceived stress, pregnancy intention, age of the respondents

*Significant at *p*-value < 0.05, 1.00—reference group

## Discussion

The objective of the present research was to assess the prevalence of depression and to identify associated factors among a sample of pregnant mothers in Ethiopia. In this study, the prevalence of depression during pregnancy was 32.3% which is suggestive of a greater probability of antenatal depression (using a PHQ9 cut-off score ≥ 8) among pregnant mothers. Some of the previous studies from Tanzania [28] and Ethiopia [29] reported an almost similar prevalence of 33.8% and 31.5%, respectively. Additional studies from Pakistan [30–32], South Africa [16, 33, 34] and Kenya [35] revealed much higher rates of prevalence of depression during pregnancy, whereas Duko et al. and Biratu and Haile [36, 37] from Ethiopia, Ogbo et al. from Australia [38] and Thompson and Ajayi from Nigeria [39] observed much lower rates of prevalence of antenatal depression. These variations in the prevalence of depression during pregnancy could be attributed to the difference in the study setting, socio-demographic characteristics and the tools used to screen depression among pregnant mothers.

Pregnant women with a low level of income were 3.19 times more likely to develop depression during pregnancy when compared to their counterparts. This is also supported by findings from other studies [23, 39, 40] reporting that the risk of depression during pregnancy will be greater in pregnant mothers with a low level of income. For example, a population-based cohort study conducted in the Netherlands that included a total of 5398 pregnant mothers to investigate the association between maternal socio-economic position and depression reported a statistically significant association between depression during pregnancy and income [21]. Further, a study that investigated the influence of early childhood and early adolescent cumulative socio-economic adversity on the occurrence of depression using a sample of 12,424 has shown a similar association [41].

Unplanned pregnancy increased the odds of depression during pregnancy. The probability of having depression during pregnancy is greater given an unplanned pregnancy [30, 35]. Epidemiological evidence suggests that planned pregnancies are linked with reduced adverse pregnancy and birth outcomes. For example, a prospective cohort study conducted to investigate pregnancy intention of 4244 pregnant women in Malawi reported that having planned pregnancies were linked with minimal risk of depression in women [42]. This is also supported by a community-based study that showed a similar result [43]. An unplanned pregnancy may result from non-use of contraceptive services, contraceptive failure and maybe from rape [44]. These women will develop depression as a result of stress which is linked with the transition into unintended motherhood and minimal social support received from her partner. However, some of the previous studies did not report such associations [45, 46]. Moreover, strengthening the counseling service and increasing access and availability of modern contraceptive methods may reduce the rates of unplanned pregnancy in Ethiopia and play a significant role in alleviating a resultant depression.

The previous history of abortion was found to have a more significant influence on maternal depression during pregnancy when compared with those who did not have a previous history of abortion. Previous studies also reported similar associations [13, 23, 44]. For example, a study that investigated the association between abortion and risk of subsequent depression in pregnant women showed that women with a history of abortion were more likely to have depression (AOR = 3.5; 95% CI 2.06.1) [47]. In a comprehensive literature review of 2018, abortion has been associated with higher rates of mental health problems when compared to women without a history of abortion [48, 49]. Further, evidence from a prospective cohort study also showed a 30% relative increase in the rate of depression among women who reported a history of abortion when compared to those who did not [50]. Pregnancy after an abortion may be psychologically and emotionally distressing. As a result, women who experienced abortion may worry about not being able to conceive or losing the current pregnancy, and this may result in depression during the current pregnancy.

## Conclusion

The prevalence of depression during pregnancy in the West Shoa zone, Oromia regional state, Ethiopia, is high at 32.3%. Correlates of antenatal depression were identified, and these were low monthly income, unplanned pregnancy and previous history of abortion. Strengthening the counseling service as well as increasing access and availability of modern contraceptive methods may reduce the rates of unplanned pregnancy in Ethiopia and this, in turn, plays a significant role in alleviating a resultant depression. Further, the integration of mental health services with the existing maternal health care was warranted to reduce depression during pregnancy in West Shoa zone health facilities of Ethiopia.

## Limitations of the study

We used a cross-sectional study design and a temporal relationship cannot be inferred using such a design. Usually, pregnant mothers start their antenatal follow-up at 1216 weeks of gestation in Ethiopia. As a result, it was so difficult to recruit pregnant mothers in their 1st trimester of pregnancy. Hence, we recruited pregnant mothers in the 2nd and 3rd trimesters. However, epidemiologic evidence suggests the intergenerational transmission of depression begins as early as the initiation of conception. As a result, the inclusion of pregnant mothers in 2nd and 3rd trimesters may underestimate the prevalence of depression in the current study. However, we adjusted for the previous history of depression to check its association with the current depression. There was also the chance of recall bias since we have assessed depression once at 2nd or 3rd trimesters. Further, we used the average monthly income to investigate the individual income level. This could underestimate the wages assessment.

## Data Availability

The dataset pertaining to this study will be shared upon reasonable request.

## Abbreviations

EDPS: Edinburgh Postnatal Depression Scale
PHQ: Patient Health Questionnaire
PSS: Perceived stress scale
WHO: World Health Organization
